# Surgical Results and Prognostic Factors of Anterior Cervical Corpectomy and Fusion for Ossification of the Posterior Longitudinal Ligament

**DOI:** 10.1371/journal.pone.0102008

**Published:** 2014-07-07

**Authors:** Yu Chen, Lili Yang, Yang Liu, Haisong Yang, Xinwei Wang, Deyu Chen

**Affiliations:** Department of Spine Surgery, Changzheng Hospital, Second Military Medical University, Shanghai, China; Toronto Western Hospital, Canada

## Abstract

**Background:**

Mechanism of ossification of the posterior longitudinal ligament (OPLL) has not been elucidated clearly. Surgical decompression is usually necessary for the patients with neurological symptoms. Anterior decompression and resection of OPLL seems to be a radical surgical option, because the spinal cord is compressed from the anterior direction.

**Methods:**

Among 229 patients who underwent ACF for OPLL between January 2001 and December 2007 in our hospital, a total of 133 patients responded to the invitation and made return visits, with a follow-up rate of 58.1%. For these patients, clinical data were collected from medical and operative records. Neurological status were evaluated by using the Japanese Orthopedic Association (JOA) scoring system. Radiological evaluations including C2-7 lordotic angle, sagittal vertical axis (SVA), occupying rate of OPLL, double-layer sign and high-intensity zone were obtained from all the patients. Complications and causes of revision surgery were also investigated. Correlations between the long-term surgical outcome and various prognostic factors were statistically analyzed.

**Findings:**

Eighty-four males and forty-nine females completed the follow-up, with a mean age at operation of 56.8 years. The overall average JOA score significantly increased, with a mean recovery rate of 64.1%±14.2%. The mean C2-7 lordotic angle and SVA were also significantly improved, and fusion rate was satisfactory. The incidence of complications was consistent to the previous reports and most of them were controllable by suitable treatments. Multiple regression analysis showed that number of corpectmies and preoperative JOA score were important predictors of surgical outcome.

**Conclusions:**

ACF is a reliable and effective method for treating OPLL patients in terms of neurological recovery, maintenance of radiological parameters, fusion rate and complications. Number of corpectomies and preoperative JOA score are important predictors for the clinical outcome when this procedure is used.

## Introduction

Since it was first reported in 1960, ossification of the posterior longitudinal ligament (OPLL) has been recognized as a common cause of cervical myelopathy in Japan [1]. Recently, it has become more and more common in China with the advances in imaging technology, such as computerized tomography [2], [3]. Although research on OPLL has progressed much in genetics and bone cell physiology, the process of ossification in this condition has never been elucidated clearly [4]. Thus, surgical decompression is usually necessary for the OPLL patients with neurological symptoms.

Surgical strategies for cervical myelopathy due to OPLL can be divided into two approaches: anterior and posterior. Anterior decompression and resection of OPLL seems to be a radical surgical option, because the spinal cord is compressed from the anterior direction [5], [6]. Yamaura et al. initially reported the direct removal method of OPLL, and then modified the procedure to anterior flotation of the OPLL, which minimized surgical invasion and decreased the risk of complications, such as hemorrhage, cerebrospinal fluid leakage and neurological deterioration [7]. However, other surgeons reported that posterior decompression was required postoperatively in 8% of the patients who underwent anterior floating procedure due to postoperative progression of OPLL [8].

Beginning from 2001, anterior corpectomy and fusion (ACF) has been chosen as the main method to treat cervical OPLL in our hospital. To achieve more adequate and lasting decompressive effect, we removed the OPLL unless it was associated with dural ossification and difficult to be separated. To assess the safety and effectiveness of this procedure, and determine the prognostic factors relevant for patients with OPLL, we conducted a retrospective study of these patients who underwent ACF for the treatment of OPLL with a minimum 5-year follow-up.

## Materials and Methods

### Patient selection and preoperative evaluation

This study was approved by the Institutional Review Broad of Second Military Medical University, Shanghai, China. Between January 2001 and December 2007, a total of 229 patients underwent anterior corpectomy and fusion (ACF) for cervical ossification of the posterior longitudinal ligament (OPLL) by two senior surgeons (DC and XW) in our department. The diagnosis of OPLL was confirmed by X-ray photographs and computer tomography, showing significant ossification behind posterior border of vertebral body. The cases, in which ossification lesion located on intervertebral levels and was difficult to be distinguished from osteophytes, were not included in this study. We chose to perform ACF for the treatment of OPLL, when it did not exceed 4 intervertebral levels (maximum 3-level corpectomies). Otherwise, we used posterior laminoplasty or laminectomy with fixation for the cases with longer extent, which were either not included in this study. In addition, the exclusion criteria in this study included trauma, neoplasm, infection, congenital deformations, previous surgery of the cervical spine and chronic system illness such as rheumatoid arthritis and neurodegenerative diseases.

All patients were clinical and radiographically evaluated before surgery. Clinical evaluation consisted of medical history and physical examination. The clinical results were assessed with the Japanese Orthopedic Association (JOA) scoring system for cervical myelopathy. Standard anterior-posterior and lateral X-rays in a standing position, computed tomography (CT) and magnetic resonance imaging (MRI) of the cervical spine were conducted as preoperative radiological evaluation. On the lateral X-ray, C2-7 lordotic angle measured from the inferior C2 endplate to the superior C7 endplate was used to evaluate cervical lordosis. C2-7 sagittal verticle axis (SVA), the horizontal distance between the posterosuperior corner of C7 and plumbline of C2 barycenter, was used to indicate cervical sagittal balance. Occupying rate (OR) was defined as the thickness of OPLL divided by the antero-posterior diameter of the bony spinal canal on the CT axial image. Double-layer sign characterized by anterior and posterior rims of hyperdense ossification separated by a central hypodense was investigated to indicate dural ossification (DO). MRI showed compression of the spinal cord and presence of high intensity zone (HIZ) in the spinal cord.

### Surgical technique

**Figure 1:**
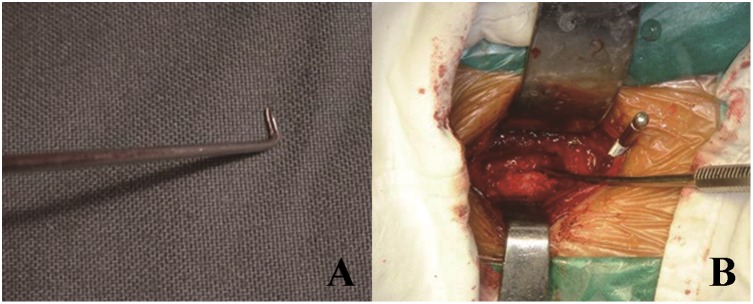
Separating OPLL from the dural mater using a specific microdissector. (A). A picture of specific microdissector. (B). Intraoperative picture.

The operation was performed via a standard right-side anterior approach. The appropriate surgical level was confirmed by intraoperative radiography. After necessary discectomies, the vertebral bodies were partially removed using an appropriate rongeur. The residual vertebral bodies and OPLL were removed by high speed drill until the OPLL was thinned as much as we can. Then, the OPLL was separated from dural mater using a specialized microdissector. The head of this dissector was a hook with a narrow slot. It was inserted under the OPLL from the nonossified ligament, rotated and slightly lifted. The ligament was cut off by scalpel along the slot. After that, the OPLL was meticulously separated using the microdissector and removed by 1–2 mm Kerrison rongeur and microcurettes (Figure 1). In this procedure, the first key step was to find the nonossified ligament, the weak part of OPLL. It was easy to be separated and cut off. Second, we used the specialized microdissector to separate OPLL from dural mater. It avoided using Kerrison rougeur directly, and was helpful to decrease the risk of CSF leakage and spinal cord injury.

**Figure 2:**
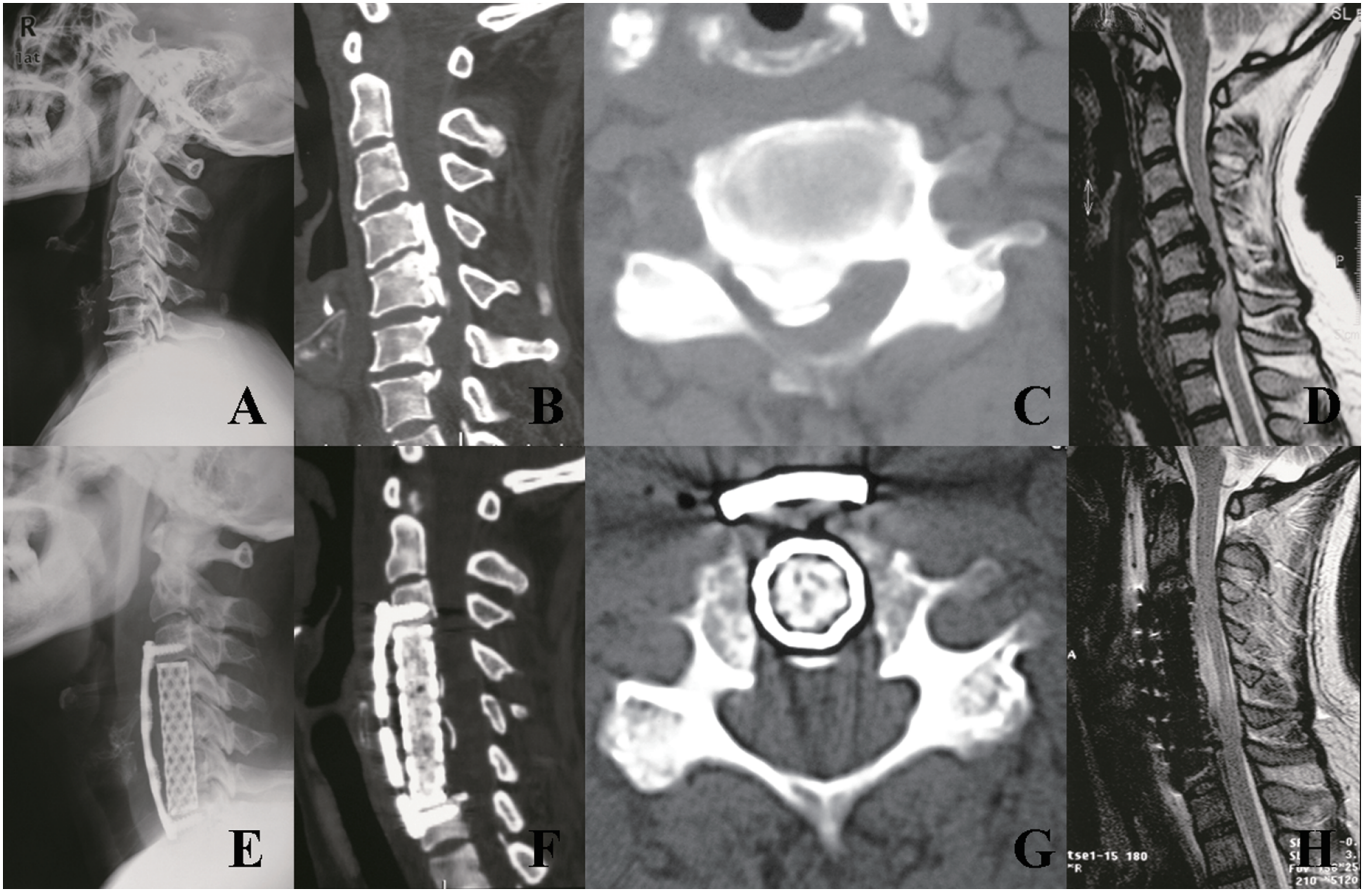
A 53-year-old men with a C4–C7 mixed-type OPLL associated with DO was treated by anterior cervical corpectomy and fusion. (A). Preoperative radiographic image at neutral position. (B, C). Preoperative sagittal and axial CT scans demonstrating a C4–C7 mixed-type OPLL with double-layer sign. (D). Preoperative MR image showed severe compression of the spinal cord. (E). Postoperative radiographic image showing C4–C6 corpectomy and fusion. (F, G). Postoperative sagittal and axial CT demonstrating complete resection of OPLL and floating of DO. (H). Postoperative MR image showed sufficient decompression of the spinal cord.

If the patient was found in the association with DO and there was a typical double-layer sign on CT scan, we tried to separate OPLL from DO through a thin layer consisting a nonossified degenerated ligament between the OPLL and the DO. This technique preserved the ossified portion of the dural mater to avoid dural defect and CSF leakage. Otherwise, the floating method was used, in which the OPLL was only separated from the around vertebral wall, but remained together with DO. After decompression, a titanium mesh cage filled with autologous bone fragments and anterior cervical plate were used to restore the stability of the involved segments (Figure 2).

After operation, our patients were required to use Philadelphia collar for at least three months. For these patients who underwent 3-level corpectomies, stabilization with Philadelphia collar was elongated to six months. If we found any significant evidence of cage subsidence or possibility of instrument failure during the follow-up period, posterior instrumentation would be considered as complemental treatment.

### Postoperative follow-up

All patients were requested to come back for a routinely examination at 3 months, 6 months and 12 months after the operation, then at irregular intervals depending on their clinical status and suggestions of the senior surgeons. These patients received invitations for clinical and radiological examinations between January and December 2013.

The examinations were performed by investigators who had no therapeutic relationship to the individual patient. Fifty-seven patients were lost to follow-up due to change of address or telephone number, 35 patients refused to return because of the long distance and old age, or the feeling that they did not have any significant problems that required a visit, 4 patients died of unrelated causes. In the end, a total of 133 patients responded to the invitation and made return visits. The consents were in written form and the consent procedure was approved by ethics committees.

At the last follow-up, clinical examination was assessed by using the JOA scoring system. The JOA recovery rate proposed by Hirabayashi was also used: recovery rate (RR) = (postoperative JOA score - preoperative JOA score)/(17 - preoperative JOA score)×100% [9]. The neurological status of the patients at the final follow-up was used to determine clinical prognosis. Standard anterior-posterior, lateral and flexion-extension X-rays of the cervical spine were obtained from every patient. CT and MRI of the cervical spine were also required, but some patients refused the procedure because they did not feel any discomfort or progression associated with their residual symptoms. Postoperative kyphotic change was defined as a decrease in the C2-7 lordotic angle greater than 10° compared with the value immediately after operation [10]. Fusion was defined as the presence of the following features: 1) absence of radiolucent lines/area across the fusion site or around any of the screw sites; 2) presence of bridging trabeculae across the fusion site; 3) absence of motion between the spinous processes on flexion-extension X-rays [11]. If fusion was questionable, it was confirmed by sagittal reconstructive CT scan.

Other demographic factors that may affect the clinical outcome such as age, gender, preoperative duration of symptoms, smoking status, combined diabetes were all reviewed. Postoperative complications including CSF leakage, C5 palsy, neurological deterioration, hoarseness, dysphagia and instrumented failure were recorded. Moreover, causes of revision surgery for these patients were also investigated.

### Statistical methods

The paired *t* test was used to detect changes in JOA score, C2-7 lordotic angle and C2-7 SVA before surgery and at the last follow-up. The Mann-Whitney U test was used to determine whether the collected factors affect the surgical outcome. Furthermore, logistic regression analysis was conducted to determine the factors best correlating to clinical results. Analysis was performed using SPSS for Windows, Version 16.0 (SPSS, Chicago, IL), and a P value of less than 0.05 was considered significant.

## Results

### Patient characteristics

A total of 84 males and 49 females completed the follow-up, with a mean age at operation of 56.8 years (range, 27 to 78 years). Preoperative clinical symptoms included mild to severe spastic limb paresis, gait disturbance, and sphincter dysfunction. Most patients decided to have a surgical decompression due to recent neurological aggravation and no response to conservative treatment. The average duration of symptoms before surgery was 32.4±12.6 months. A smoking habit was found in 36 (27.1%) patients, and 45 (33.8%) patients were associated with diabetes before surgery. Preoperative CT scans showed the overall meaning occupying rate (OR) of OPLL reached to 48.4%±22.7%. The double-layer sign indicating dural ossification (DO) was observed in 42 (31.6%) cases. On preoperative T2-weighted MR images, high intensity zone (HIZ) was found in 61 (45.9%) cases.

According to the extent of OPLL and compression of the spinal cord, cervical corpectomy was performed at 1 level in 56 (42.1%) patients, 2 levels in 42 (31.6%) patients and 3 levels in 35 (26.3%) patients. The clinical and radiological characteristics according to numbers of corpectomies were detailed in Table 1. Statistical analysis revealed that there were no significant differences in clinical features among three groups. However, preoperative CT scan revealed that OR of OPLL and incidence of DO on in the 3-level corpectomied group were significantly higher than those in the 1 or 2-level groups. On preoperative MR images, HIZ was also found in more patients in the 3-level group.

**Table 1:** Summary of preoperative clinical features and radiological evaluation.

	1-level corpectomy	2-level corpectomy	3-level corpectomy	Total
No. of patients	56	42	35	133
Males (%)	38(67.9)	26(61.9)	20(57.1)	84(63.2)
Age (years)	51.2±6.8	57.4±7.5	58.7±8.0	56.8±6.4
Duration of symptoms (months)	28.6±14.8	34.4±20.6	35.2±22.7	32.4±12.6
Smoking status(%)	18(32.1)	12(28.6)	11(31.4)	41(30.8)
Combined with diabetes (%)	24(42.9)	17(40.5)	14(40.0)	55(41.4)
OR of OPLL	40.6±24.4	46.8±28.2	63.7±32.5*	48.4±22.7
Dural ossification (%)	14(25.0)	12(28.6)	16(45.7)*	42(31.6)
HIZ on T2-wieghted MRI (%)	22(39.3)	18(42.9)	21(60.0)*	61(45.9)

No., number, OPLL, ossification of the posterior longitudinal ligament; OR, occupying rate, HIZ, high intensity zone;

# P<0.05 when was compared with 1-level group;

*P<0.01 when was compared with 1- or 2-level groups.

### Clinical and radiological outcomes

The overall average JOA score was 9.6±1.4 preoperatively and 13.7±1.6 at the final follow-up, with a mean recovery rate of 64.1%±14.2%. The mean JOA scores were all significantly improved after operation in three groups (P<0.01), but the postoperative JOA score and recovery rate of the 3-level corpectomied group were significant lower than those of the 1- or 2-level group (P<0.05).

The overall C2-7 lordotic angle was 15.5°±5.4° preoperatively and 22.8°±6.2° at the final follow-up. The mean C2-7 lordotic angles were all significantly increased after operation in three groups (P<0.01), and there was no significant differences between three groups. The overall C2-7 SVA was significantly decreased from 35.4±14.6 mm to 31.2±13.8 mm (P<0.01). Among the three subgroups, it was improved by 1- to 2-level corpectomy (P<0.01), but no significant change by 3-level corpectomy. At the final follow-up, postoperative kyphotic change was observed at 7 (5.3%) patients, and overall fusion rate was 95.6%. The incidence of postoperative kyphotic change and fusion rate were similar between three groups (Table 2).

**Table 2:** Clinical and radiological results at the last follow-up.

	1-level corpectomy	2-level corpectomy	3-level corpectomy	Total
JOA score				
Preoperative	9.7±1.8	9.6±1.7	9.4±1.7	9.6±1.4
Postoperative	14.2±22#	13.9±2.1#	12.6±1.9# *	13.7±1.6#
RR(%)	71.4±24.7	67.1±22.4	48.5±17.6*	64.1±14.2
C2-7 lordotic angle (°)				
Preoperative	15.4±8.6	16.2±7.0	14.7±8.2	15.5±5.4
Postoperative	23.2±7.4#	22.6±7.5#	22.2±7.8#	22.8±6.2#
C2-7 SVA (mm)				
Preoperative	34.4±14.7	35.8±14.9	36.5±16.4	35.4±14.6
Postoperative	29.6±14.2#	30.8±15.3#	34.2±15.7	31.2±13.8#
Postoperative kyphotic change(%)	3(5.4)	2(4.8)	2(5.7)	7(5.3)
Fusion (%)	54(96.4)	40(95.2)	34(97.1)	128(95.6)

JOA, Japanese Orthopedic Association; OPLL, ossification of the posterior longitudinal ligament; SVA, sagittal vertical axis;

# P<0.01 when was compared with preoperative variables;

*P<0.05 when was compared with 1- or 2-level groups.

### Complications and causes of revision surgery

The complications of our patients included cerebrospinal fluid (CSF) leakage, C5 palsy, neurological deterioration, hoarseness, dysphagia and instrument failure (Table 3). Overall incidence of each complication was 8.3% (CSF leakage), 3.0% (C5 palsy), 2.3% (neurological deterioration), 8.3% (hoarseness), 11.3% (dysphagia), and 0.8% (instrument failure). Statistical analysis showed that the incidence of CSF leakage, hoarseness and dysphagia was significantly higher in the 3-level corpectomied group (p<0.01). Most complications could be cured be conservative treatments. If CSF leakage occurred after operation, the drainage tube was pulled out within 12 hours after operation and continuous pressure to the wound was applied using plastic bandage until it stopped. In our experience, it usually stopped after 1- to 2-week local-pressure treatment. The patients who developed C5 palsy and neurological deterioration were treated including neurotrophy drugs, hyperbaric oxygen therapy and functional exercises. The patients recovered after 3- to 6-month conservative treatment. Hoarseness and dysphagia mainly occurred in the early stage after operation, and gradually released during the follow-up period.

**Table 3:** Summary of complications and causes of revision surgery.

	1-level corpectomy	2-level corpectomy	3-level corpectomy	Total
Complications (%)				
CSF leakage	4(7.1)	3(7.1)	4(11.4)*	11(8.3)
C5 plasy	1(1.8)	2(4.8)	1(2.9)	4(3.0)
Neurological deterioration	1(1.8)	1(2.4)	1(2.9)	3(2.3)
Hoarseness	2(3.6)	2(4.8)	7(20.0)*	11(8.3)
Dysphagia	2(3.6)	2(4.8)	11(31.4)*	15(11.3)
Instrumented failure	1(1.8)	0(0.0)	0(0.0)	1(0.8)
Causes of revision surgery (%)				
CSF leakage	0(0.0)	1(2.4)	0(0.0)	1(0.8)
Instrument failure	1(1.8)	0(0.0)	0(0.0)	1(0.8)

CSF, cerebrospinal fluid; OPLL, ossification of the posterior longitudinal ligament;

*P<0.01 when was compared with 1- or 2-level groups.

Among these patients, only two patients (1 CSF leakage and 1 instrument failure) needed revision surgery. The first patient who underwent 2-level corpectomy developed CSF pseudocyst after operation. Her neck was still swelling after half a year, so revision surgery to repair dural matter was performed for this patient using fibrin sealant. After revision surgery, the CSF pseudocyst disappeared and wound cured better. Another patient who underwent 1-level corpectomy need revision surgery because screw breakages occurred with cage subsidence, and anterior revision surgery was performed to change screws.

### Prognostic factors

Univariate analysis revealed that gender, age, smoking status, combined diabetes, OR of OPLL, dural ossification and HIZ were not related to the surgical outcome. However, patients with longer duration of symptoms (≥24 months), more number of corpectomies (≥3), or lower preoperative JOA score (<9 points) were significantly prone to have poor surgical outcomes (Table 4). Furthermore, multiple regression analysis showed that number of corpectomies and preoperative JOA score were important predictors of surgical outcome (Table 5).

**Table 4:** Correlations of postoperative JOA score and various factors.

	No. of patients	Postoperative JOA score	P value
Gender			
Male	84	13.64±1.73	0.528
Female	49	13.80±1.92	
Age (years)			
<60	72	13.54±1.68	0.323
≥60	61	13.99±1.95	
Duration of symptoms (months)			
<24	51	14.12±2.14	0.001
≥24	82	13.44±1.83	
Smoking status			
Yes	41	13.28±2.23	0.245
No	92	13.89±1.38	
Combined with diabetes			
Yes	55	13.47±1.56	0.627
No	78	13.86±1.93	
Preoperative JOA score			
<9	48	12.80±2.43	0.000
≥9	85	14.20±1.84	
Levels of corpectomies			
1 to 2 levels	98	14.09±2.13	0.000
≥3 levels	35	12.60±1.94	
OR of OPLL (%)			
<50	74	13.84±1.84	0.345
≥50	59	13.52±1.92	
Dural ossification			
Yes	42	13.57±2.18	0.534
No	91	13.76±1.39	
HIZ on T2-wieghted MRI			
Yes	61	13.41±1.45	0.237
No	72	13.95±1.92	

JOA, Japanese Orthopedic Association; OPLL, ossification of the posterior longitudinal ligament; OR, occupying rate, HIZ, high intensity zone.

**Table 5:** Results of multiple regression analysis of included factors to predict surgical outcome.

	JOA score at the end of follow-up	
Factors	Coefficient	P value
Age	−0.104	0.265
Duration of symptom	−0.076	0.134
Smoking status	0.083	0.356
Combined with diabetes	0.016	0.843
Preoperative JOA score	0.745	0.000
Levels of corpectomies	−0.318	0.000
OR of OPLL	−0.053	0.428
Dural ossification	0.032	0.746
HIZ on T2-wieghted MRI	0.078	0.427

JOA, Japanese Orthopedic Association; OPLL, ossification of the posterior longitudinal ligament; OR, occupying rate, HIZ, high intensity zone.

## Discussion

Previous epidemiological studies have shown that the incidence of OPLL is relatively higher among Japanese people. It was reported to be 1.9%–4.3% in Japanese over age of 30 years old, significantly higher than that reported for Western populations (0.10%–1.8%) [12]–[17]. However, only a few studies have been conducted on the general Chinese. A study in Taiwan revealed the incidence of OPLL was 0.2% for the Chinese and 0.4% for the Takasago Tribe population more than 30 years of age [2]. Another study in the Northeast of China showed it was 1.6% among the Chinese and 1.8% among the Mongolians [3]. Recently, a larger epidemiological study was carried out in the Middle-east of China that involved 15673 Chinese. The result showed the overall incidence of OPLL was 2.4% among the Chinese, but it developed predominantly at middle age and the incidence increased to be 5.6% among the people more than 50 years of age [18].

Treatment of OPLL seems to be one of more and more common clinical practices for spine surgeons in China, but choice of procedure is still the subject of argument and no criteria for choosing surgical procedure have been established at this moment. In general, previous reports have not shown any significant difference in the long-term surgical outcome between the posterior and anterior procedures, but anterior decompression and fusion is technically demanding and has a higher incidence of surgery-related complications [10], [19]–[23]. This apprehension has led surgeons to avoid the anterior procedure in past years, and they tended to choose the easier and safer posterior procedure (e.g., laminoplasty). Laminoplasty provides indirect decompression by shifting the spinal cord posteriorly and leaves the OPLL. However, cases have been reported of postoperative worsening of spinal alignment and growth of the OPLL causing further deterioration of myelopathy [24], [25]. Other investigators also recommended choosing anterior decompression and fusion for the patients with the following characteristics: (1) OPLL occupying ratio of >60%; (2) hill-shipped ossification; (3) locally kyphotic alignment of the cervical spine [20], [23]. However, the policy of choosing the anterior procedure only for critical cases was objected by some authors, as this might decrease the number of spine surgeons who can perform the anterior procedure skillfully [26]. Surgeons should not choose laminoplasty just because it is easier, especially in patients for whom the anterior procedure is more suitable. Thus, we performed anterior corpectomy and fusion for the patients in whom OPLL did not exceed 4 intervertebral levels, and chose posterior laminoplasty or laminectomy with fixation for the cases with longer extent. In this procedure, we used titanium mesh cage and anterior plate for cervical restoration, which avoided donor site complications of traditional autologous bone graft. However, a high failure rate of a stand-alone anterior plate for 3-level corpectomies without posterior instrumentation has been extensively reported. Therefore, we required them to use Philadelphia collar for six months, and posterior instrumentation would be considered, when evidence of cage subsidence or possibility of instrumented failure was observed during the follow-up. However, in our patients including those who did and did not complete follow-up, only one patient with 1-level corpectomy developed instrument failure, and need revision surgery. One reason for this high success rate may be that the OPLL patients seldom have osteoporosis, which leads to cage subsidence and instrumented failure. Furthermore, property of bone formation in the OPLL patients may contribute to have a higher fusion rate. Thus, the safety and utility of titanium mesh cage and anterior plate may be different between the patients with cervical spondylotic myelopathy and those with OPLL.

Previous studies have showed favorable results were achieved by direct anterior decompression. In one of the largest published surgical series, Mizuno and Nakagawa reported their experience in treating 107 patients with OPLL who underwent anterior cervical corpectomy and direct reovmal of the ossified mass. Patients underwent follow-up for 6 months. 89% of 104 patients who presented with myelopathy improved, and all three patients who presented with radiculopathy improved [5]. Recently, Odate et al. reported the surgical outcome of anterior corpectomy and fusion (ACF) combined with an anterior cervical plate for treating OPLL in 68 patients with an average follow-up of 29.6 months. The average recovery rate of JOA score was 63.0% ±32.3% [26]. The strength of this study is that a larger patient population with at least 5-year follow-up may provide insights into the long-term outcome of ACF in the treatment of patients with OPLL. However, a relatively low follow-up rate of 58.1% was a significant weakness of this study. To make up this weakness, we also reviewed the patients who did not complete the follow-up and summarized their clinical features, complications and surgical results in Table 6. These patients were mostly treated by 1- to 2-level corpectomy. No more perioperative complications developed compared with the patients who completed 5-year follow-up in this study. Furthermore, the early surgical results were also relatively better at the final follow-up (range 6–54 months, mean 26.4 months). These information would be helpful for readers to appreciate the safety and utility of this approach. The results of this study showed that ACF was generally effective, with a significantly improved JOA score and the average recovery rate reached to be 64.1%±14.2%. Long-term results of radiographic follow-up showed ACF was effective in maintenance of cervical lordosis and sagittal balance. Bone fusion was also satisfactory, and was not correlated with number of corpectomies.

**Table 6:** Clinical features, complications and surgical results of other 96 patients who did not complete the last follow-up in this study.

	All patients (n = 96)
Males (%)	52(54.2)
Age (years)	52.4±7.2
Duration of symptoms (months)	28.7±15.2
Smoking status (%)	28(29.2)
Combined with diabetes (%)	35(36.5)
OR of OPLL	42.6±17.4
Number of corpectomies (%)	
1-level	57(59.4)
2-level	35(36.5)
3-level	4(4.2)
Complications (%)	
CSF leakage	6(6.3)
C5 palsy	2(2.1)
Neurological deterioration	0(0.0)
Hoarseness	5(5.2)
Dysphagia	8(8.6)
JOA score	
Preoperative	9.7±1.9
Postoperative (final follow-up)	15.2±1.8
RR (%)	74.6±19.6

No., number, OPLL, ossification of the posterior longitudinal ligament; OR, occupying rate, HIZ, high intensity zone; CSF, cerebrospinal fluid; JOA, Japanese Orthopedic Association.

The main complications in this study included CSF leakage, C5 palsy, neurological deterioration, hoarseness, dysphagia, and instrumented failure, which was consistent to the previous studies [27], [28]. The overall complication rate in this study was not higher, but more CSF leakage, hoarseness and dysphagia occurred in our patients, especially in the 3-level corpectomied group. Hoarseness and dysphagia after cervical anterior approach may result from endotracheal intubation, laryngeal edema or spasm, symptomatic hematoma, or injury to the laryngeal vagus nerve. They mainly occurred in the early stage after operation and recovered by themselves. With regard to CSF leakage, some authors thought that it was the result of the evolution of OPLL because the dura adhered by progressive OPLL was easily torn. In addition, it should be noted that dural ossification (DO) around OPLL also easily caused CSF leakage, which resulted from the primary ossified dura that was worn away by a chronic pressure and abrasion phenomenon. It is very helpful for spine surgeons to recognize DO via CT scan preoperatively and use microsurgery technique to separate OPLL from the DO as we have reported in surgical technique [29], [30].

As previously reported, the surgical outcomes were not always good. Several factors had been evaluated in other studies, including age, sex, ossification type, duration of preoperative symptoms, involved levels, diabetes, preoperative neurological score, and related radiographic parameters. Some factors were confirmed as predictors [31]–[35]. It seemed that age at surgery was related to the surgical outcome of the posterior approach rather than the anterior approach [32]. Among the imaging measurements, the transverse area of the spinal cord, ROM of the cervical spine, and HIZ in T2-wieghted MRI were also confirmed to correlate with the recovery rate of the posterior approach [33], [34]. With regard to anterior approach, only diabetes was proved to be related with surgery of OPLL in one study [35]. Our research first revealed that postoperative JOA score was correlated with number of corpectomies. Neurological result was relatively worse in the 3-level corpectomied group compared with those in the 1- and 2-level groups. Preoperative radiographic assessments suggested the patients in the 3-level corpectomied group had bigger ossification and more severe compression of the spinal cord, which consequently resulted in a lower postoperative JOA score. Furthermore, preoperative JOA score was also confirmed to be correlated with the surgical outcome by multiple regression analysis. The JOA score is devised for patients with cervical myelopathy and provides a semi-quantitative assessment of functions by evaluating the ability to eat, ambulate, and void. In this study, it was proved to be a relative accurate prognostic factor for predicting surgical outcome of the patients with OPLL.

In conclusion, based on a minimum 5-year follow-up of ACF for the patients with OPLL, the results were satisfactory in terms of postoperative JOA score, recovery rate, maintenance of cervical lordosis and sagittal balance. Complications were also controllable with suitable treatments. Statistical analysis showed that number of corpectomies and preoperative JOA score were significantly related to the surgical outcomes, which should be highlighted in the preoperative communication with patients.
